# Low body mass index as a predictor of sputum culture conversion and treatment outcomes among patients receiving treatment for multidrug-resistant tuberculosis in Lesotho

**DOI:** 10.1080/16549716.2024.2305930

**Published:** 2024-02-02

**Authors:** Lawrence Oyewusi, Chengbo Zeng, KJ Seung, Stephanie Mpinda, Mikanda Kunda, Carole D Mitnick, Makelele Kanu, Meseret Tamirat, Joalane Makaka, Mabatloung Mofolo, Refiloe Maime, Llang Maama, Ninza Senyo, Bamidele Oguntoyinbo, Lwayi Mayombo, Molly F Franke

**Affiliations:** a Clinical department (MDRTB), Partners In Health, Maseru, Lesotho; b Department of Global Health and Social Medicine, Harvard Medical School, Boston, MA, USA; c Division of Global Health Equity, Brigham and Women’s Hospital, Boston, MA, USA; d National TB and Leprosy Programme, Lesotho Ministry of Health, Maseru, Lesotho

**Keywords:** Lesotho, MDRTB, BMI, new drugs, culture conversion

## Abstract

**Background:**

A low body mass index (BMI) at the start of treatment for rifampicin- or multidrug-resistant tuberculosis (MDR/RR-TB) is associated with poor treatment outcomes and may contribute to delayed sputum culture conversion, thereby prolonging the period of potential transmission to others. Whether the relative importance of low BMI in predicting treatment outcomes differs by HIV status is unclear.

**Objectives:**

We evaluated the association between low BMI and two dependent variables, sputum culture conversion and end-of-treatment outcome, among patients receiving treatment for MDR/RR-TB in Lesotho, a setting with a high prevalence of HIV infection.

**Methods:**

Secondary data from a prospective cohort of patients initiating a longer (18–20 months) treatment containing bedaquiline and/or delamanid under routine programmatic conditions in Lesotho were analysed. Risk ratios and differences were adjusted for potential confounders using multivariable logistic regression, and estimates were stratified by HIV status.

**Results:**

Of 264 patients, 105 and 250 were eligible for culture conversion and end-of-treatment analyses, respectively. Seventy-one per cent of patients (74/105) experienced culture conversion within six months, while 74% (184/250) experienced a favourable end-of-treatment outcome. Low BMI was associated with a lower frequency of culture conversion at six months among those who were not living with HIV (relative risk [RR]: 0.50 [95% CI: 0.21, 0.79]); this association was attenuated among those living with HIV (RR: 0.88 [95% CI: 0.68, 1.23]). A low BMI was moderately associated with a lower frequency of treatment success (RR = 0.89 [95% CI: 0.77, 1.03]), regardless of HIV status.

**Conclusions:**

Low BMI was common and associated with the frequency of six-month culture conversion and end-of-treatment outcomes. The association with culture conversion was more pronounced among those not living with HIV. Addressing the myriad factors that drive low BMI in this setting could hasten culture conversion and improve end-of-treatment outcomes. This will require a multipronged approach focused on alleviating food insecurity and enabling prompt diagnosis and treatment of HIV and TB.

## Background

Despite the recent improvements in the treatment of multidrug- or rifampicin-resistant and resistant tuberculosis (MDR/RR-TB), not all patients experience treatment success [1,2]. Even among patients receiving all-oral and/or shortened regimens, those living with HIV or advanced TB experience a higher risk of unfavourable treatment outcomes. Low body mass index (BMI) indicates advanced disease and may therefore correlate with unsuccessful treatment [3]. Low BMI is also a proxy for malnutrition, including micronutrient and protein energy deficiencies, which may impact immune system functioning and, thereby, the response to treatment [4]. Because malnutrition and advanced TB may occur simultaneously or singularly, the underlying cause of the low body weight may be challenging to disentangle. Regardless of the mechanism, low BMI may serve as a marker of risk or vulnerability, identifying patients needing additional care and support.

Sputum culture conversion is a marker of treatment response; delayed or no culture conversion identifies patients at high risk for an unfavourable end-of-treatment outcome [5,6]. Studies from diverse settings, including two meta-analyses, have suggested that low BMI is an important prognostic factor for delayed culture conversion and poor end-of-treatment outcomes among patients undergoing treatment for MDR/RR-TB [3,7–13]. While some of these studies were conducted in settings with a high HIV prevalence [10,12], whether the relative importance of low BMI differs according to HIV status with regard to these outcomes remains unclear.

Lesotho is a small, landlocked country surrounded by the Republic of South Africa. The country’s population is estimated at 2.2 million, with 57% living on less than one dollar a day and about 80% living in rural areas [14]. TB incidence is 650 per 100,000 population, and the estimated proportion of cases that are MDR/RR-TB is 11.8% across new and previously treated cases [15]. In Lesotho, the tuberculosis epidemic is fuelled by HIV (one in five adults aged 15 to 49 lives with HIV) [16]. It co-occurs alongside frequent malnutrition and food insecurity arising from El Niño-induced drought. Over 57% of the population lives below the poverty line [14].

Given the synergistic epidemics of tuberculosis, HIV, food insecurity, and poverty in Lesotho, low BMI may constitute a particularly important warning sign for patients who present to care for MDR/RR-TB. Among patients treated for MDR/RR-TB with a longer regimen containing bedaquiline and/or delamanid, we examined whether a low BMI predicted culture conversion within six months of treatment or end-of-treatment success and whether these relationships were modified by HIV status.

## Methods

### Data resource, target population, and study sites

This secondary analysis relied on data from the prospective endTB observational cohort (NCT03259269) of patients initiating longer (18–20 month) treatment for MDR/RR-TB with a regimen containing bedaquiline and/or delamanid under routine programmatic conditions. Data from standardised forms were entered into an electronic medical record. The current analysis was restricted to patients from Lesotho who had documented MDR/RR-TB and consented to participate in the observational cohort. For analyses of culture conversion, we additionally required a positive baseline culture taken before the initiation of treatment [17,18]. For the end-of-treatment (EOT) outcome analyses, patients were excluded if their EOT outcome was not evaluated (i.e. in the case of transfer out of the country). All patients in Lesotho received a monthly food package including 50 kg of maize meal, 20 kg of sorghum, 2 kg of beans, 2 kg of peas, 1 g of sugar, 5 kg of vegetable oil, and 1 g of powdered milk. This support was intended for one person and provided to enable and encourage treatment adherence and improve patients’ nutritional status.

### Dependent variables and definitions

Sputum culture conversion within the first six months of treatment was defined as two consecutive negative cultures collected at least 15 days apart, one before 180 days of treatment and one before 210 days of treatment [19–21]. The second outcome was favourable EOT outcomes. In accordance with WHO guidelines, using an algorithm, we calculated the EOT outcomes based on the available culture results at the end of treatment [22].

### Independent variables and definitions

The primary independent variable was BMI, which was classified into underweight (<18.5), normal weight (18.5 to 24.9), and overweight (≥25). We considered potential confounding by demographic characteristics (i.e. age and sex), substance use (i.e. alcohol use, tobacco use, injection drug use, and non-injection drug use), comorbidities (i.e. anaemia, HIV infection, diabetes mellitus or glucose intolerance, hepatitis B virus, hepatitis C virus), and TB-related characteristics (i.e. bilateral disease, cavitary disease, fibrosis, sputum culture, sputum smear, and baseline resistance profile).

*Statistical analysis* included descriptive statistics, bivariable analyses, and multivariable logistic regression. From the list of potential confounders identified *a priori*, we identified those associated with BMI and the outcomes of interest in these data and adjusted for these potential confounders. Besides the list of potential confounders, we adjusted for resistance profile at baseline and numbers of likely effective Group A drugs in the multivariable analyses. We created an interaction term between BMI and HIV status to examine whether the impact of BMI on treatment outcome differed by HIV status. The missing indicator method was used to handle missing data. In the case of model nonconvergence, we used bootstrapping with 500 random samples to calculate the risk ratio (RR), risk difference (RD), and 95% confidence intervals (CI) based on the predicted probabilities.

We conducted a secondary analysis to differentiate between underweight and severely underweight. For the analysis of culture conversion, we conducted a sensitivity analysis, which consisted of a time-to-event analysis in which we censored patients who died or became lost to follow-up before the six months of treatment. Analyses were conducted using SAS software version 9.4 (SAS Institute, Inc., Cary, NC), and the forest plot was created using the ‘forestplot’ package in R version 4.1.0 (The R Foundation).

### Research ethics

The study protocol for the endTB observational cohort was approved by the ethics review committees for the three consortium partners (i.e. Partners In Health, Medecins Sans Frontieres, and Interactive Research and Development) and in each country where the study was conducted. In Lesotho, approval was granted by the National Health Research Ethics Committee (REF: 11; 61-2016; March 2015). Study participants provided written informed consent for inclusion in the observational cohort.

## Results

### Overview

Of the 2,789 patients enrolled in the endTB observational cohort, 264 (9%) were from Lesotho. A total of 105 and 250 patients were eligible for culture conversion analyses and end-of-treatment analyses, respectively. Seventy-one per cent of patients (74/105) experienced culture conversion within six months, while 74% (184/250) shared a favourable EOT outcome. Across both analytic groups, patients tended to be male and married. More than half were underweight, and a majority were living with HIV (Table 1).

**Table 1.** Baseline characteristics of patients initiating treatment for rifampicin- or multidrug-resistant tuberculosis in Lesotho, 2015–2018.

Variables	Culture conversion (*N* = 105)		EOT outcome (*N* = 250)	
	Available sample	n (%)	Available sample	n (%)
Age at study drug start (year, median, IQR)	105 (100)	43 (34, 57)	250 (100)	41 (32, 55)
Sex, Female	105 (100)	32 (31)	250 (100)	94 (38)
Alcohol use	99 (94)	22 (22)	238 (95)	47 (20)
Tobacco use	101 (96)	15 (15)	239 (96)	30 (13)
Married or living with a partner	103 (98)	58 (56)	244 (98)	142 (58)
BMI at baseline	103 (98)		234 (94)	
Underweight		53 (51)		113 (48)
Normal		40 (39)		99 (42)
Overweight		10 (10)		22 (9)
**Comorbidities at baseline**				
Anaemia^Δ^	102 (97)	76 (75)	239 (96)	173 (72)
HIV infection	105 (100)	80 (76)	250 (100)	196 (78)
CD4 count ≤200 cells/µl	72 (69)	43 (60)	170 (68)	99 (58)
Viral load ≤1000 copies/ml	59 (56)	28 (47)	124 (50)	57 (46)
On ART at treatment start date	80 (76)	59 (74)	196 (78)	145 (74)
Time on ART before treatment start date (year, median, IQR)	59 (56)	3 (0, 8)	145 (58)	1 (0, 6)
Diabetes mellitus*	86 (72)	17 (20)	203 (81)	23 (11)
Hepatitis B infection	100 (95)	9 (9)	234 (94)	17 (7)
Hepatitis C infection	100 (95)	0 (0)	238 (95)	1 (0)
At least one other comorbidities	105 (100)	10 (10)	250 (100)	20 (8)
**TB-related characteristics at baseline**				
Bilateral disease	62 (59)	49 (79)	137 (55)	99 (72)
Cavitary disease	61 (58)	22 (36)	128 (51)	41 (32)
Fibrosis	61 (58)	34 (56)	128 (51)	65 (51)
Culture positive	105 (100)	105 (100)	170 (68)	101 (59)
Sputum smear positive	99 (94)	50 (50)	180 (72)	60 (33)
Resistance profile	105 (100)		250 (100)	
MDR/RRTB without additional resistance FQ/INJ		54 (51)		88 (35)
MDR/RRTB without testing to FQ and INJ		23 (22)		118 (47)
MDR/RRTB + FQ or INJ resistance		21 (20)		28 (11)
MDR/RRTB + FQ and INJ both resistance		7 (7)		11 (4)
Not tested for MDR/RR		–		5 (2)
Previous treatment	105 (100)		248 (99)	
None		48 (46)		122 (49)
1^st^ line		40 (38)		91 (36)
2^nd^ line		17 (16)		35 (14)
**Use of BDQ and DLM at baseline**	105 (100)		250 (100)	
BDQ (without DLM)		37 (35)		96 (38)
DLM (without BDQ)		65 (62)		146 (58)
Both BDQ and DLM		3 (3)		8 (3)
Total numbers of likely effective drugs (Median, IQR)	105 (100)	5 (5, 6)	250 (100)	5 (5, 6)
Numbers of likely effective drugs in Group A (Median, IQR)	105 (100)	1 (1, 2)	250 (100)	1 (1, 2)

IQR: Interquartile range; BDQ: Bedaquiline; DLM: Delamanid; EOT: End-of-treatment.

*Diabetes mellitus was defined by self-report, random blood sugar >200 mg/dl or 11.1 (mmol/L), or HbA1c ≥6.5.

^Δ^To assess anaemia, we considered haemoglobin results taken up to 30 days after treatment initiation. The haemoglobin thresholds for anaemia were <12 g/dl for females and <13 g/dl for males.

### Culture conversion within six months

**Figure 1.**
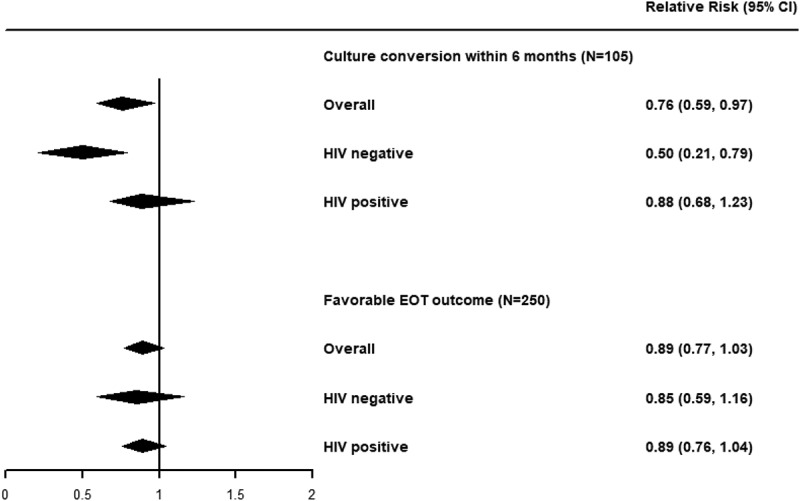
Forest plot of the adjusted relative risk of low BMI on culture conversion within six months or favourable EOT outcomes, stratified by HIV status.

In univariable analyses, being underweight (crude RR = 0.75 [95% CI]: 0.58, 0.99]; Table A1 in Appendix) was associated with a lower probability of culture conversion. Adjusting for potential confounders (i.e. age, marital status, anaemia, HIV infection, resistance profile at baseline, and numbers of likely effective Group A drugs), being underweight was associated with a similar reduction in the probability of culture conversion within six months (RR: 0.76 [95% CI: 0.59, 0.97]; RD: −0.19 [95% CI: −0.34, −0.03]; Table 2, Figure 1).

RR: Relative risk. CI: Confidence interval. EOT: End-of-treatment.

**Table 2.** Multivariable analyses of low BMI and culture conversion and favourable treatment outcome among patients initiating treatment for rifampicin- or multidrug-resistant tuberculosis in Lesotho, 2015–2018.

	Culture conversion within six months (*N* = 105)†			Favourable EOT outcomes (*N* = 250)‡		
	Proportion (%)	Relative risk (95%CI)	Risk difference (95%CI)	Proportion (%)	Relative risk (95%CI)	Risk difference (95%CI)
**Model 1 (no interaction)**						
BMI at baseline						
Underweight	32 (60)	0.76 (0.59, 0.97)	−0.19 (−0.34, −0.03)	79 (70)	0.89 (0.77, 1.03)	−0.09 (−0.19, 0.02)
Normal	32 (80)	Reference		78 (79)	Reference	
Overweight	8 (80)	1.01 (0.69, 1.36)	0.01 (−0.26, 0.26)	19 (86)	1.08 (0.92, 1.28)	0.07 (−0.07, 0.22)
**Model 2 (interaction by HIV)**						
*HIV negative*						
BMI at baseline						
Underweight	6 (50)	0.50 (0.21, 0.79)	−0.50 (−0.79, −0.21)	15 (65)	0.85 (0.59, 1.16)	−0.11 (−0.35, 0.11)
Normal	11 (100)	Reference		19 (76)	Reference	
Overweight	2 (100)	1.00 (1.00, 1.00)	0.00 (0.00, 0.00)	4 (80)	1.03 (0.47, 1.48)	0.02 (−0.40, 0.32)
*HIV positive*						
BMI at baseline						
Underweight	26 (63)	0.88 (0.68, 1.23)	−0.08 (−0.25, 0.15)	64 (71)	0.89 (0.76, 1.04)	−0.09 (−0.20, 0.03)
Normal	21 (72)	Reference		59 (80)	Reference	
Overweight	6 (75)	1.05 (0.57, 1.58)	0.03 (−0.34, 0.36)	15 (88)	1.10 (0.92, 1.29)	0.08 (−0.06, 0.22)

CI: Confidence interval. EOT: End-of-treatment.

**†**Adjusted for age, marital status, anaemia, HIV infection, resistance profile at baseline, and numbers of likely effective Group A drugs.

**‡**Adjusted for age, HIV infection, diabetes, at least one other comorbidity, bilateral disease, cavitary disease, fibrosis, positive sputum culture at baseline, resistance profile at baseline, and numbers of likely effective Group A drugs.

We found potential evidence for effect modification by HIV status. Among patients without HIV, being underweight was associated with a sizeable reduction in the frequency of six-month culture conversion (RR: 0.50 [95% CI: 0.21, 0.79]; RD: −0.50 [95% CI: −0.79, −0.21]; Table 2, Figure 1). This relationship was attenuated for patients living with HIV (RR: 0.88 [95% CI: 0.68, 1.23]; RD: −0.08 [95% CI: −0.25, 0.15]; Table 2, Figure 1).

In the secondary analyses, which differentiated between underweight and severely underweight, the results were consistent and did not affect the interpretation (Table A2 in Appendix). Being severely underweight (RR: 0.78 [95% CI: 0.57, 1.01]; RD: −0.18 [95% CI: −0.37, 0.01]) or underweight (RR: 0.78 [95% CI: 0.57, 1.01]; RD: −0.18 [95% CI: −0.36, 0.00]) was negatively associated with culture conversion within six months. Among patients who were HIV negative, being severely underweight (RR: 0.52 [95% CI: 0.00, 1.00]; RD: −0.48 [95% CI: −1.00, 0.00]) or underweight (RR: 0.49 [95% CI: 0.00, 0.83]; RD: −0.52 [95% CI: −1.00, −0.17]) was associated with a reduction in the risk of six-month culture conversion. This association was less pronounced in patients living with HIV (severely underweight: RR: 0.89 [95% CI: 0.60, 1.23]; RD: −0.08 [95% CI: −0.31, 0.14]; underweight: RR: 0.93 [95% CI: 0.64, 1.33]; RD: −0.05 [95% CI: −0.27, 0.20]).

Sensitivity analyses, in which patients who died or were lost to follow-up before six months of the treatment were censored, yielded similar results. Patients who were underweight experienced a lower rate of culture conversion overall (HR: 0.44 [95% CI: 0.26, 0.78]) (Table A3 in Appendix); however, this impact tended to be stronger among patients who were HIV-negative (HR: 0.20 [95% CI: 0.07, 0.56]) as compared to those living with HIV (HR: 0.60 [95% CI: 0.31, 1.14]).

### Favourable EOT outcomes

In univariable analyses, being underweight (crude RR: 0.89 [95% CI: 0.76, 1.04]) or overweight (crude RR: 1.10 [95% CI: 0.90, 1.33]; Table A4 in Appendix) was not associated with the probability of favourable outcomes.

In multivariable analyses adjusted for age at study drug start, HIV infection, diabetes, at least one other comorbidity, bilateral disease, cavity disease, fibrosis, and positive culture at baseline, we found moderate evidence that patients with TB who were underweight were less likely to have a favourable outcome after treatment (RR: 0.89 [95% CI: 0.77, 1.03]; RD: −0.09 [95% CI: −0.19, 0.02]). The impact of being underweight was similar among those who were (RR: 0.85 [95% CI: 0.59, 1.16]; RD: −0.11 [95% CI: −0.35, 0.11]) and were not living with HIV (RR: 0.89 [95% CI: 0.76, 1.04]; RD: −0.09 [95% CI: −0.20, 0.03]) (Table 2, Figure 1).

The results of secondary analyses were also consistent with the main findings (Table A5 in Appendix). We found that patients who were severely underweight (RR: 0.79 [95% CI: 0.66, 0.94]; RD: −0.17 [95% CI: −0.29, −0.04]) or underweight (RR: 0.92 [95% CI: 0.77, 1.08]; RD: −0.07 [95% CI: −0.19, 0.06]) at baseline suffered from a lower probability of a favourable outcome. These results were similar, regardless of HIV status.

## Discussion

Low BMI was common in this cohort of patients treated for MDR/RR-TB in Lesotho, most of whom also lived with HIV. Low BMI was strongly associated with a lower frequency of culture conversion at six months and moderately associated with a lower frequency of treatment success. This is similar to a study by Park et al., where low BMI was an independent risk factor for failure to achieve sputum culture conversion within three months among MDR/RR-TB patients [2,8,23]. While the impact of low BMI on favourable EOT outcomes appeared to be similar among those who were and were not living with HIV, low BMI seemed to be a more important predictor of culture conversion among patients who were not living with HIV than those living with HIV.

The high prevalence of low BMI in this population suggests that targeted interventions that effectively prevent and/or mitigate this condition could impact culture conversion and treatment success. The development of such interventions requires understanding the factors driving low BMI in this setting, which are likely multifactorial. These include food insufficiency and advanced HIV and/or TB disease, all common among patients treated for MDR/RR-TB in Lesotho. A large part of the Basotho population experiences chronic food insecurity and/or nutrient deficiency, especially residents of rural areas where subsistence farming is common despite unfavourable agricultural conditions [24,25]. Advanced HIV infection and delayed TB diagnosis or treatment may also be important contributors. In Lesotho, migration in pursuit of economic opportunities is common and may interfere with consistent antiretroviral treatment in the absence of differentiated programmes to ensure mechanisms for treatment retention [26,27]. Traditional beliefs may contribute to tuberculosis treatment delays in Lesotho as some patients prefer to seek healthcare from traditional healers first and only present at the hospital when their clinical conditions have significantly deteriorated [28]. Traditional healers have been successfully engaged to promote HIV care in Lesotho and could likewise be engaged to facilitate prompt care for tuberculosis [29]. Health system challenges and centralised services may also contribute to delayed linkage to care and treatment initiation. Based on Lesotho’s national drug-resistant TB guidelines, all bacteriologically confirmed drug-resistant TB patients should be referred to a specialised hospital for treatment initiation [30,31]. Centralised approaches may lead to delays along the care cascade, resulting in morbidity, mortality, and ongoing TB transmission [32,33]. An approach where care and treatment are decentralised to the district hospitals where patients are initiated on treatment once diagnosed may be a solution to delayed treatment initiation among MDR/RR-TB patients in the country. As a result, the country is currently implementing a decentralisation strategy for the care and treatment of all drug-resistant TB patients.

The finding that low BMI was particularly harmful for individuals not living with HIV was unexpected. Rather, we hypothesised that low BMI could be more harmful in the context of HIV due to impaired immune functioning. While this unanticipated finding may be due to chance, an alternative explanation is that low BMI reflects social and medical histories that may vary by HIV status. For example, low BMI may be more likely to represent advanced TB disease among individuals without HIV than those living with HIV. A low BMI resulting from advanced HIV may be less likely than a low BMI resulting from advanced TB disease to impact TB-related outcomes. A second example is that patients with HIV may be more likely than those without HIV to have low BMI due to food insufficiency (and, therefore, may be more likely to respond to monthly nutritional support packages provided during TB treatment) [34]. The major limitation of this analysis was an inability to discern between different contributors to low BMI. Future analyses could incorporate markers of food insecurity, recent weight loss, and/or qualitative in-depth interviews to elucidate the prevailing causes of low BMI and identify promising interventions.

Among the other limitations of this analysis is the lack of information on baseline HIV control and its correlates (i.e. adherence to antiretroviral therapy, time on antiretroviral therapy) which may influence BMI and TB treatment outcome. Additionally, while we hypothesise that treatment delays may have contributed to the high prevalence of low BMI at baseline, we did not have data on the onset of symptoms to confirm this hypothesis.

In conclusion, low BMI was common among patients treated with MDR/RR-TB and appeared to impact the frequency of six-month culture conversion and favourable EOT outcomes. Addressing the multiple factors that drive low BMI in this setting will require a multipronged approach focused on alleviating food insecurity and enabling prompt diagnosis and treatment of HIV and TB to hasten culture conversion and improve end-of-treatment outcomes.

## Paper context

A low BMI at the start of treatment for rifampicin- or multidrug-resistant tuberculosis (MDR/RR-TB) is associated with poor treatment outcomes and may contribute to late sputum culture conversion, thereby prolonging the period of potential transmission to others. Whether the relative importance of low BMI in predicting treatment outcomes differs by HIV status is unclear. Given the synergistic epidemics of tuberculosis, HIV, food insecurity, and poverty in Lesotho, low BMI may constitute a particularly important warning sign for patients who present to care for MDR/RR-TB. Among patients treated for MDR/RR-TB with a longer regimen containing bedaquiline and/or delamanid, we examined whether a low BMI predicted culture conversion within six months of treatment or end-of-treatment success and whether these relationships were modified by HIV status.

## Appendix

**Table A1.** Univariate logistic regressions of baseline variables and culture conversion within six months among patients initiating treatment for rifampicin- or multidrug-resistant tuberculosis in Lesotho, 2015–2018 (*N* = 105).

Variables	Culture converged (%)	RR (95%CI)	*p*-value
**Total**	74 (71)	-	-
Age at study drug start (year, median, IQR)	41.5 (32, 56)	0.99 (0.98, 1.00)	**0.028***
Sex, Female	24 (75)	1.10 (0.85, 1.42)	0.484
Alcohol use	16 (73)	1.04 (0.77, 1.40)	0.809
Tobacco use	11 (73)	1.03 (0.74, 1.45)	0.845
Non injection drug use	2 (67)	0.95 (0.42, 2.17)	0.906
Married or living together	38 (66)	0.84 (0.66, 1.08)	0.170*
BMI at baseline			
Underweight	32 (60)	0.75 (0.58, 0.99)	**0.041***
Normal	32 (80)	Reference	
Overweight	8 (80)	1.00 (0.70, 1.42)	0.999
**Comorbidities at baseline**			
Anaemia	52 (68)	0.85 (0.66, 1.08)	0.182*
HIV infection	55 (69)	0.91 (0.69, 1.18)	0.461
CD4 counts ≤200 cells/µl	29 (67)	1.03 (0.73, 1.45)	0.866
Viral load ≤1000 copies/ml	21 (68)	1.00 (0.70, 1.43)	0.993
Diabetes (Yes)	10 (59)	0.80 (0.52, 1.22)	0.291
Hepatitis B infection (Yes)	7 (78)	1.07 (0.74, 1.56)	0.713
At least one other comorbidities (Yes)	7 (70)	0.99 (0.65, 1.53)	0.973
**TB-related characteristics at baseline**			
Bilateral disease	33 (67)	1.09 (0.68, 1.76)	0.709
Cavity disease	14 (64)	0.96 (0.65, 1.41)	0.814
Fibrosis	22 (65)	0.97 (0.67, 1.40)	0.873
Smear sputum			
Negative	39 (80)	Reference	
Scanty & 1+	13 (72)	0.91 (0.66, 1.25)	0.553
2+	9 (69)	0.87 (0.59, 1.29)	0.484
3+	11 (58)	0.73 (0.48, 1.10)	0.130
Resistance profile			
RR/MDRTB no additional resistance FQ/INJ	38 (70)	Reference	
RR/MDRTB no testing to FQ and INJ	16 (70)	0.99 (0.71, 1.37)	0.944
RR/MDRTB + FQ or INJ resistance	17 (81)	1.15 (0.88, 1.51)	0.312
RR/MDRTB + FQ and INJ both resistance	3 (43)	0.61 (0.25, 1.47)	0.268
Numbers of effective drugs in Group A at baseline	1 (1, 2)	1.04 (0.86, 1.26)	0.682

BMI: Body mass index. RR: Relative risk. CI: Confidence interval. *Potential confounders with *p*-value less than 0.20.

**Table A2.** Multivariate analysis of four-group BMI and culture conversion within six months among patients initiating treatment for rifampicin- or multidrug-resistant tuberculosis in Lesotho, 2015–2018 (*N* = 105).

	Proportion (%)	Relative risk (95%CI)	Risk difference (95%CI)
**Model 1 (without interaction)**			
BMI at baseline			
Severely underweight	14 (58)	**0.78 (0.57, 1.01)**	**−0.18 (−0.37, 0.01)**
Underweight	18 (62)	**0.78 (0.57, 1.01)**	**−0.18 (−0.36, 0.00)**
Normal	32 (80)	Reference	
Overweight	8 (80)	1.01 (0.69, 1.37)	0.01 (−0.27, 0.27)
**Model 2 (with interaction)**			
*HIV negative*			
BMI at baseline			
Severely underweight	2 (50)	**0.52 (0.00, 1.00)**	**−0.48 (−1.00, 0.00)**
Underweight	4 (50)	**0.49 (0.00, 0.83)**	**−0.52 (−1.00, −0.17)**
Normal	11 (100)	Reference	
Overweight	2 (100)	1.00 (1.00, 1.00)	0.00 (0.00, 0.00)
*HIV positive*			
BMI at baseline			
Severely underweight	12 (60)	0.89 (0.60, 1.23)	−0.08 (−0.31, 0.14)
Underweight	14 (67)	0.93 (0.64, 1.33)	−0.05 (−0.27, 0.20)
Normal	21 (72)	Reference	
Overweight	6 (75)	1.05 (0.56, 1.58)	0.04 (−0.34, 0.37)

BMI: Body mass index. CI: Confidence interval. Potential confounders related to both culture conversion and low BMI at baseline (e.g. age, marital status, anaemia, HIV infection, resistance profile at baseline, numbers of effective drugs in Group A) were adjusted.

**Table A3.** Multivariate Cox regression of BMI and culture conversion within six months among patients initiating treatment for rifampicin- or multidrug-resistant tuberculosis in Lesotho, 2015– 2018 (*N* = 105).

Variables	HR (95% CI)
**Model 1 (without interaction)**	
BMI at baseline	
Underweight	**0.44 (0.26, 0.78)**
Normal	Reference
Overweight	1.01 (0.42, 2.44)
**Model 2 (with interaction)**	
*HIV negative*	
BMI at baseline	
Underweight	**0.20 (0.07, 0.56)**
Normal	Reference
Overweight	1.15 (0.13, 10.00)
*HIV positive*	
BMI at baseline	
Underweight	0.60 (0.31, 1.14)
Normal	Reference
Overweight	1.08 (0.41, 2.82)

BMI: Body mass index. HR: Hazard ratio; CI: Confidence interval. Potential confounders related to both culture conversion and low BMI at baseline (e.g. age, HIV infection, resistance profile at baseline, numbers of likely effective drugs in Group A) were adjusted in the analysis.

**Table A4.** Univariate logistic regressions of baseline variables and favourable EOT outcome among patients initiating treatment for rifampicin- or multidrug-resistant tuberculosis in Lesotho, 2015–2018 (*N* = 250).

Variables	Favourable outcome (%)	RR (95%CI)	*p*-value
**Total**	**184 (74)**	-	-
Age at study drug start (year, median, IQR)	39 (30, 52.5)	0.99 (0.99, 1.00)	**<0.001***
Sex, Female	71 (76)	1.04 (0.90, 1.21)	0.586
Alcohol use	37 (79)	1.08 (0.91, 1.29)	0.372
Tobacco use	22 (73)	0.98 (0.78, 1.24)	0.880
Injection drug use	2 (67)	0.90 (0.40, 2.02)	0.797
Non injection drug use	4 (80)	1.08 (0.69, 1.69)	0.740
Married or living together	101 (71)	0.93 (0.80, 1.08)	0.346
BMI			
Underweight	79 (70)	0.89 (0.76, 1.04)	0.140*
Normal	78 (79)	Reference	–
Overweight	19 (86)	1.10 (0.90, 1.33)	0.357
**Comorbidities at baseline**			
Anaemia	125 (72)	0.92 (0.78, 1.07)	0.277
HIV infection	146 (75)	1.06 (0.87, 1.28)	0.561
CD4 counts ≤200 cells/µl	77 (78)	1.06 (0.89, 1.27)	0.503
Viral load ≤1000 copies/ml	48 (72)	0.87 (0.72, 1.05)	0.154*
Diabetes	15 (65)	0.82 (0.60, 1.11)	0.194*
Hepatitis B infection	13 (77)	0.98 (0.75, 1.29)	0.896
At least one other comorbidities	11 (55)	0.73 (0.49, 1.10)	0.129*
**TB-related characteristics at baseline**			
Bilateral disease	62 (63)	0.77 (0.62, 0.95)	**0.016***
Cavity disease	23 (56)	0.78 (0.57, 1.05)	0.097*
Fibrosis	40 (62)	0.84 (0.66, 1.08)	0.171*
Culture positive	66 (65)	0.73 (0.62, 0.86)	**<0.001***
Smear sputum			
Negative	95 (79)	Reference	
Scanty & 1+	17 (74)	0.93 (0.72, 1.21)	0.605
2+	11 (73)	0.93 (0.67, 1.28)	0.638
3+	15 (68)	0.86 (0.64, 1.16)	0.330
Resistance profile			
RR/MDRTB no additional resistance FQ/INJ	60 (68)	Reference	
RR/MDRTB no testing to FQ and INJ	90 (76)	1.12 (0.94, 1.33)	0.210
RR/MDRTB + FQ or INJ resistance	20 (71)	1.05 (0.80, 1.38)	0.740
RR/MDRTB + FQ and INJ both resistance	10 (91)	1.33 (1.05, 1.69)	**0.017***
Not tested for RR/MDRTB	4 (80)	1.17 (0.74, 1.87)	0.497
Numbers of effective drugs in Group A (Median, IQR)	1 (1, 2)	1.01 (0.91, 1.13)	0.815

BMI: Body mass index. RR: Relative risk. CI: Confidence interval. EOT: end-of-treatment. *Potential confounders with *p*-value less than 0.20.

**Table A5.** Multivariate analysis of four-group BMI and favourable EOT outcome among patients initiating treatment for rifampicin- or multidrug-resistant tuberculosis in Lesotho, 2015–2018 (*N* = 250).

	Proportion (%)	Relative risk (95%CI)	Risk difference (95%CI)
**Model 1 (without interaction)**			
BMI at baseline			
Severely underweight	32 (67)	**0.79 (0.66, 0.94)**	**−0.17 (−0.29, −0.04)**
Underweight	47 (72)	**0.92 (0.77, 1.08)**	**−0.07 (−0.19, 0.06)**
Normal	78 (79)	Reference	
Overweight	19 (86)	1.08 (0.92, 1.28)	0.07 (−0.07, 0.22)
**Model 2 (with interaction)**			
*HIV negative*			
BMI at baseline			
Severely underweight	4 (67)	0.75 (0.24, 1.30)	−0.19 (−0.62, 0.23)
Underweight	11 (65)	0.85 (0.56, 1.20)	−0.12 (−0.36, 0.14)
Normal	19 (76)	Reference	
Overweight	4 (80)	1.03 (0.47, 1.48)	0.02 (−0.39, 0.32)
*HIV positive*			
BMI at baseline			
Severely underweight	28 (67)	**0.79 (0.64, 0.95)**	**−0.17 (−0.30, −0.04)**
Underweight	36 (75)	0.94 (0.78, 1.11)	−0.05 (−0.19, 0.09)
Normal	59 (80)	Reference	
Overweight	15 (88)	1.10 (0.93, 1.29)	0.08 (−0.06, 0.22)

BMI: Body mass index. CI: Confidence interval. EOT: End-of-treatment. Potential confounders related to both favourable EOT outcome and low BMI at baseline (e.g. age, HIV infection, diabetes, at least one comorbidity, bilateral disease, cavity disease, fibrosis disease, positive sputum culture at baseline, resistance profile at baseline, numbers of effective drugs in Group A) were adjusted.
